# Reviewer acknowledgement 2013

**DOI:** 10.1186/1916-0216-43-3

**Published:** 2014-02-25

**Authors:** Hadi Seikaly, Erin Wright

**Affiliations:** 1 University of Alberta, Alberta, Canada

## Abstract

**Contributing reviewers:**

The Editors of *Journal of Otolaryngology - Head & Neck Surgery* would like to thank all our reviewers who have contributed to the journal in Volume 42 (2013).

## 

Don Anderson

Canada

Kal Ansari

Canada

Melissa Avelino

Brazil

Claus Bachert

Belgium

Brittany Barber

Canada

Brian Blakley

Canada

Michael Brandt

Canada

Lawrence Brecht

United States of America

Shamir Chandarana

Canada

Jason Chau

Canada

Gabriela Constantinescu

Canada

Martin Corsten

Canada

David Cote

Canada

Jean Davidson

Canada

Chris Diamond

Canada

Scott Durham

Canada

Peter Dziegielewski

United States of America

Hamdy El-Hakim

Canada

Wael El-Matary

Canada

Jeremy Freeman

Canada

Michael Friedman

United States of America

Kevin Fung

Canada

Richard Gall

Canada

Lesley Garber

Canada

Gerald Grant

United States of America

Menachem Gross

Israel

Michael Gupta

Canada

Robert Hart

Canada

David Hatcher

United States of America

Eric Henry

Canada

Michael Hier

Canada

Allan Ho

Canada

Paul Hong

Canada

Robert Irvine

Canada

Amin Javer

Canada

Athanasios Katsarkas

Canada

Paul Kerr

Canada

Haytham Kubba

United Kingdom

Boyd Lee

Canada

John Lee

Canada

Vincent Lin

Canada

Yuan-Chi Lin

United States of America

Anthony Magit

United States of America

John Manoukian

Canada

Emad Massoud

Canada

Wayne Matthews

Canada

Laurie McLean

Canada

Corey Moore

Canada

Anthony Nichols

Canada

Dan O'Connell

Canada

Flordeliz Osler

Canada

Richard Payne

Canada

Magdy Riad

Egypt

Keith Richardson

Canada

Matthew Rigby

Canada

Brian Rotenberg

Canada

Kathryn Roth

Canada

Doron Sommer

Canada

Leigh Sowerby

Canada

Chris Szeto

Canada

S. Mark Taylor

Canada

Jamie Tibbo

Canada

Jean-Philippe Vaccani

Canada

Allan Vescan

Canada

Harissios Vliagoftis

Canada

Richard Voegels

Brazil

Ezekiel Weis

Canada

Brian Westerberg

Canada

John Yoo

Canada

